# Screening of Lignocellulose-Degrading Superior Mushroom Strains and Determination of Their CMCase and Laccase Activity

**DOI:** 10.1155/2014/763108

**Published:** 2014-02-12

**Authors:** Li Fen, Zhu Xuwei, Li Nanyi, Zhang Puyu, Zhang Shuang, Zhao Xue, Li Pengju, Zhu Qichao, Lin Haiping

**Affiliations:** ^1^The Nurturing Station for the State Key Laboratory of Subtropical Silviculture, Provincial Engineering Laboratory of Biopesticide Preparation, Zhejiang A&F University, Lin'an 311300, China; ^2^School of Agricultural and Food Science, Zhejiang A&F University, Lin'an 311300, China; ^3^Qingyuan Forestry Bureau of Zhejiang, Qingyuan 323800, China

## Abstract

In order to screen lignocellulose-degrading superior mushroom strains ten strains of mushrooms (*Lentinus edodes939*, *Pholiota nameko*, *Lentinus edodes868*, *Coprinus comatus*, *Macrolepiota procera*, *Auricularia auricula*, *Hericium erinaceus*, *Grifola frondosa*, *Pleurotus nebrodensis*, and *Shiraia bambusicola*) were inoculated onto carboxymethylcellulose agar-Congo red plates to evaluate their ability to produce carbomethyl cellulase (CMCase). The results showed that the ratio of transparent circle to mycelium circle of *Hericium erinaceus* was 8.16 (*P* < 0.01) higher than other strains. The filter paper culture screening test showed that *Hericium erinaceus* and *Macrolepiota procera* grew well and showed extreme decomposition of the filter paper. When cultivated in guaiacol culture medium to detect their abilities to secrete laccase, *Hericium erinaceus* showed the highest ability with the largest reddish brown circles of 4.330 cm. CMCase activity determination indicated that *Coprinus comatus* and *Hericium erinaceus* had the ability to produce CMCase with 33.92 U/L on the 9th day and 22.58 U/L on the 10th day, respectively, while *Coprinus comatus* and *Pleurotus nebrodensis* had the ability to produce laccase with 496.67 U/L and 489.17 U/L on the 16th day and 18th day. Based on the results, *Coprinus comatus* might be the most promising lignocellulose-degrading strain to produce both CMCase and laccase at high levels.

## 1. Introduction

Lignocellulose is an abundant and renewable source of carbohydrates that can be converted into value-added products in the earth. It is estimated that 6.0 × 10^9^ tons of lignocellulose are generated by photosynthesis each year all over the world, but only about 20% are used for conversion into energy and food [1]. Many lignocellulose litters such as straws and sawdusts are produced each year in industry and agriculture. Therefore it is of great importance to make these wastes available to reduce environmental pollution and stabilize the development of bioenergy [2].

Agricultural residues are rich in lignocellulosic compounds whose disposal and handling are problematic due to their complex structure and decomposition properties [3, 4]. Lignin wraps around cellulose and hemicelluloses fibers, which inhibits the degradation of both, so it is necessary to remove the lignin to facilitate the use of cellulose and hemicelluloses as a source for bioenergy. Recently, chemical and physical methods of pretreating lignocellulosic compounds have been used to expose the underlying cellulose and hemicelluloses such as radicalization, steam explosion, puffing, acid, and alkali [5]. Unfortunately, these methods consume high amounts of energy and cause pollutants [6]. So it is pressing to find a high-efficiency, energy-saving, and environment-friendly way to break down cellulose and hemicelluloses.

Some microorganisms, especially some edible and medicinal mushrooms, produce a complete set of enzymes capable of efficient degradation of native cellulose and lignin, so screening of lignocellulose-degrading superior mushroom strains has become a significant project in the process of reusing lignocellulose wastes. Enzymes which are responsible for cellulose degradation are hydrolytic [7]. The cellulose-hydrolysing enzymes (i.e., cellulases) are divided into three major groups: endoglucanases, cellobiohydrolases (exoglucanases), and *β*-glucosidases [8, 9]. Lignin degradation mainly depends on oxidative enzymes [10], including lignin peroxidase, manganese-dependent peroxidase, and laccase which are well studied. In wood-destroying white rot fungi, laccases are the most important components of the lignolytic complex responsible for decomposition of lignin, which is one of the most-difficult-to-be-decomposed polymers in nature [11, 12].

Many researches focus on the fields of screening of lignocellulose-degrading superior strains, while many others focus on high production mushrooms all over the world, but very few put the two areas together. At present, microbes used in cellulose degradation are mainly fungi, of which *Trichoderma*, *Aspergillus*, *Rhizopus*, and *Myrothecium* have been studied the most [3]. Since edible and medicinal fungi can convert lignincellulose to form fruiting bodies, it indicated they can decompose lignocellulose to micromolecular nutrient substance for fungi to be utilized. Thus studies on screening of lignocellulose-degrading superior edible fungi play an important role [13]. In this study 10 strains of mushrooms were investigated for their ability to degrade lignocellulose. The purpose and significance of the research are obtaining some strains of mushrooms that have high ability to degrade lignocellulose. So in the process of cultivating them on lignocellulose litters, mushroom production and raw material to produce bioenergy could be obtained at the same time.

## 2. Materials and Methods

### 2.1. Fungal Strains and Culture Media

Ten commercial strains (*Lentinus edodes939* ACCC51655, *Pholiota nameko* ACCC51896, *Lentinus edodes868* ACCC51717, *Coprinus comatus* ACCC51773, *Macrolepiota procera* ACCC51531, *Auricularia auricula* ACCC51604, *Hericium erinaceus* ACCC51322, *Grifola frondosa* ACCC51962, *Pleurotus nebrodensis* ACCC51498, and *Shiraia bambusicola* ACCC50027 (Agricultural Culture Collection of China)) were subjected to screening experiments.

All strains were seeded on potato dextrose agar medium (PDA: potato 200 mg/mL glucose 20 mg/mL and agar 20 mg/mL) slants and discs and incubated at 26°C for 7–10 days until colonies appeared which were prepared for the following experiments.

Carboxymethylcellulose (CMC)-agar culture medium contained 10 mg/mL sodium CMC, 4 mg/mL (NH_4_)_2_SO_4_, 2 mg/mL KH_2_PO_4_ and 20 mg/mL agar [14], in which sodium carboxymethylcellulose (CMC_Na_) was the sole carbon source for the fungus.

Filter paper culture medium contained 1 mg/mL (NH_4_)_2_SO_4,_ 1 mg/mL KH_2_PO_4_, 0.7 mg/mL MgSO_4_·7H_2_O, 0.5 mg/mL NaCl, and 6 cm × 1 cm filter paper [14, 15].

CMCase production medium contained 10 mg/mL   CMC_Na_, 4 mg/mL (NH_4_)_2_SO_4_, 2 mg/mL KH_2_PO_4_, 0.5 mg/mL MgSO_4_·7H_2_O, 10 mg/mL peptone, and 5 mg/mL beef extract [3].

Guaiacol color culture medium contained 1.0 mg/mL guaiacol, 0.1 mg/mL C_4_H_12_N_2_O_6_, 2.6 mg/mL peptone, 0.5 mg/mL MgSO_4_·7H_2_O, 1 mg/mL KH_2_PO_4_, 0.2 mg/mL Na_2_HPO_4_, and 20 mg/mL agar [16].

Laccase production medium was equal to PDA without agar.

All media were autoclaved at 121°C for 20 minutes.

### 2.2. CMC Culture Screening

Five mm disc diameter was taken from the PDA plates as inoculated on CMC plates and then grew at 26°C for about one week until mycelium was proper to measure. Afterwards, the plates were dyed by adding 20 mL Congo red (1 mg/mL), and three hours later, 1 mol/L NaCl was added to decolorize them. The diameters of transparent circle and mycelium circle on each plate were measured.

The ratio of transparent circle to colony diameter was then calculated to estimate the ability of the fungi to degrade cellulose [17]. The experiment was repeated three times. The data presented in Table 1 represent mean values ± standard deviation.

### 2.3. Filter Paper Culture Screening

Each 15 × 150 mm test tube was filled with 5 mL filter paper culture medium so that there for sure was 1 cm of 6 × 1 cm filter paper out of the liquid. Strains which were inoculated into the filter paper culture medium were kept at the surface of the liquid and in the filter paper and then cultivated statically at 26°C for about half a month. The experiment was repeated three times.

### 2.4. Guaiacol Color Culture Screening

**Figure 1:**
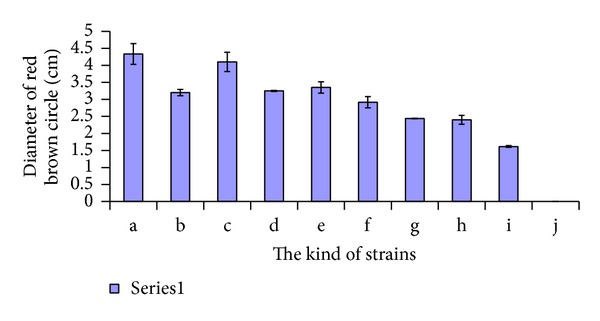
Diameter of red brown circle of 10 strains. One week after inoculating on guaiacol color culture media. a: *Hericium erinaceus*, b: *Coprinus comatus*, c: *Lentinus edodes868*, d: *Lentinus edodes939*, e: *Pleurotus nebrodensis*, f: *Auricularia auricular*, g: *Pholiota nameko*, h: *Macrolepiota procera*, i: *Shiraia bambusicola*, and j: *Grifola frondosa*. The data represent the average of three independent experiments.

One mycelium agar disc (five mm diameter), cut from the growing edge of each strain PDA culture, was used to inoculate each plate and incubated at 26°C for about one week and then monitored for the appearance of a reddish brown circle. The diameter of the reddish brown circle was measured to identify laccase secreting ability of the stains. The experiment was repeated three times. The data presented in Figure 1 represent values ± standard deviation.

### 2.5. Fermentation

Superior cellulose degradation strains were selected based on plate culture results and inoculated into 250 mL Erlenmeyer flasks containing 50 mL of CMCase production medium. Five mycelium agar discs (five mm diameter) cut from the growing edge of each strain 7-day PDA culture were used to inoculate each flask. The strains were cultivated in a rotary shaker at 120 rev·min^−1^ at 26°C and were sampled each day for five days. All experiments were performed with three replicates.

### 2.6. Enzyme Assay

**Figure 2:**
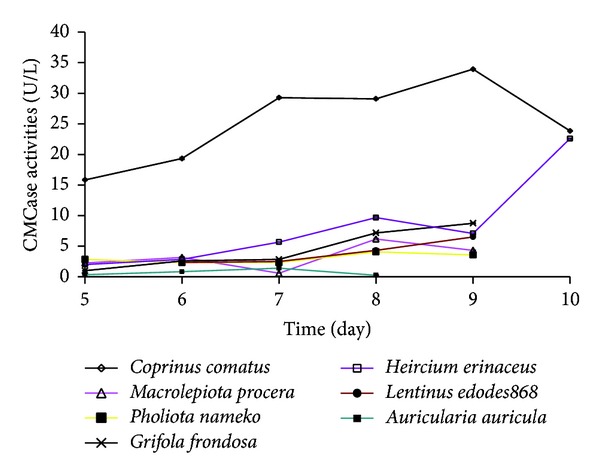
Time courses of CMCase activity of 7 strains. Five mycelium agar discs (5 mm diameter) were used as the inoculum. Cultivated in CMC liquid medium, 1 mL Zymotic fluid of the strains was taken out of the flask for experiment each day. The data represent the average of three independent experiments.

Carboxymethylcellulase (CMCase) was estimated by using 1% carboxymethyl cellulose as the sole carbon source. The reaction mixture was composed of 0.2 mL enzyme sample and 1.8 mL CMC (1%) dissolved in 0.1 mol/L sodium acetate buffer (pH 4.8). The reaction was kept at 50°C for 30 minutes. The amount of reducing sugar was determined by the DNS(3,5-dinitrosalicylic) method [18], using glucose as a standard at 550 nm. As a control, 1.8 mL of sodium acetate buffer was used instead of CMC following the same treatment [19]. Enzymatic activity, expressed as U/mL, was defined as the amount of enzyme producing 1 *μ*g of product per minute per mL of substrate extracted. Each same experiment was repeated three times. The data in Figure 2 represent the average of three independent experiments.

**Figure 3:**
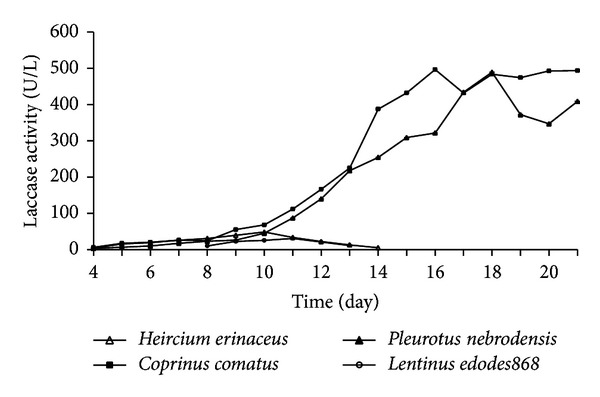
Time courses of laccase activity of 4 strains. Five mycelium agar discs (5 mm diameter) were used as the inoculum. Cultivated in potato dextrose liquid medium, 1 mL Zymotic fluid of the strains was taken out of the flask for experiment each day. The data represent the average of three independent experiments.

The strains which showed the capacity to secrete laccase in guaiacol color culture screening were used to determine laccase activity and were conducted in 3 mL reaction mixtures consisting of 2.7 mL of 0.1 mol/L sodium acetate buffer (pH 4.5), 0.2 mL of 1 mmol/L 2,20′-azinobis(3-ethylbenzothiazoline-6-sulfonic acid) (ABTS) solution, and 0.1 mL extracellular culture supernatant. The reaction was monitored by measuring the change in 420 nm for 3 min. One unit of enzyme activity is defined as the amount of oxidation of ABTS followed by 0.1 increase in absorbance at 420 nm. The molar absorption coefficient of oxidized ABTS is 3.6 × 10^4^ L/(mol·cm) [1]. Each same experiment was repeated three times. The data in Figure 3 represent the average of three independent experiments.

## 3. Results

### 3.1. CMC Culture and Filter Paper Culture Screening

Mushrooms were cultivated in CMC-agar culture to determine their ability to use CMC_Na_ as the sole carbon source. All strains tested showed different mycelial growth in CMC culture. Based on estimations of their mycelium diameters, *Shiraia bambusicola* grew best with mycelium growth diameter of 5.91 cm, followed by *Pholiota nameko*,* Lentinus edodes868*, and *Lentinus edodes939*, where mycelium growth diameters were 5.04 cm, 3.84 cm, and 3.49 cm, respectively. *Hericium erinaceus* had the smallest mycelium growth diameter of 0.61 cm followed by *Auricularia auricular* with 0.62 cm mycelium diameter. However, a rather different case was observed in the transparent circles that represent cellulose degradation. *Shiraia bambusicola* and *Pleurotus nebrodensis* showed no transparent circle, and *Auricularia auricula* with 1.41 cm transparent circle became the third inferior candidate, while *Pholiota nameko* followed by *Hericium erinaceus* had the largest transparent circles (5.18 cm and 4.95 cm, resp.). To sum up, *Hericium erinaceus* had the highest ratio of transparent circle diameter to mycelium circle diameter (*d1*/*d2*) with the datum of 8.16. Big differences were observed among *d1*/*d2* of 10 different strains (Table 1).

The filter paper culture screening test showed that all strains grew slowly with evidence of mycelium extension in approximately half a month. The growth results also varied from strain to strain. *Hericium erinaceus* and *Macrolepiota procera* grew well in the filter paper culture medium and showed extreme decomposition of the filter paper, while *Grifola frondosa*, *Shiraia bambusicola*, and *Coprinus comatus* showed scarce biomass growth and without observable degradation. Some strains such as *Lentinus edodes868* and *Macrolepiota procera* showed less than optimal growth but were still able to decompose the filter paper. All strains were divided into four groups according to the degree of decomposing the filter paper. *Hericium erinaceus* and *Macrolepiota procera* decomposed the filter paper in a good manner. Most of the strains decomposed the filter paper ordinarily, including *Lentinus edodes868*, *Pleurotus nebrodensis*, *Lentinus edodes939*, *Auricularia auricula*, and* Coprinus comatus*. While *Grifola frondosa* and *Pholiota nameko* faintly decomposed the filter paper, *Shiraia bambusicola* showed no degradation ofthe filter paper (Table 2). As shown in Tables 1 and 2, the results of CMC culture and filter paper culture screening experiment are approximately consistent.

### 3.2. Guaiacol Color Culture Screening

Guaiacol is one of the substrates of laccase and the degradation product of guaiacol is rufous. Therefore we used the red brown color to indicate the laccase production by the strains and the diameter of the colored circle to estimate the quantity of laccase produced. All plates showed a reddish brown color after one week except for the plate with *Grifola frondosa* which was reddish brown the second day after inoculation. As time went by, the reddish brown circle expanded and eventually stabilized. *Hericium erinaceus*, *Lentinus edodes868*, *Pleurotus nebrodensis*, and *Coprinus comatus* had large reddish brown circles. Their reddish brown circle diameters were 4.33 cm, 4.10 cm, 3.35 cm, and 3.20 cm, respectively (Figure 1). So these four strains were selected to determine the activity of laccase.

### 3.3. Determination of CMCase Activity

Based on the quantitative analysis of transparent circles to mycelial growth diameter, we were more interested in the numerical value of enzyme activity. The determination showed that CMCase activity of *Coprinus comatus* was obviously higher than others. As the incubation time increased, CMCase activity also increased and the peak (33.92 U/L) appeared on day 9. *Hericium erinaceus* showed the second highest CMCase activity. The variation rule of CMCase activity of *Hericium erinaceus* seemed similar to *Coprinus comatus*. However, the activity produced two peaks: one on day eight and the other on day 10. Furthermore, the CMCase activity increased dramatically on day 10 by nearly threefold more than that on day nine and 2.34-fold of the first peak. *Pholiota nameko* showed no significant CMCase activity increase during the cultivation. Although CMCase activity of *Grifola frondosa* and *Macrolepiota procera* gradually increased, the level of activity remained low. It seemed that *Auricularia auricular* was deficient in CMCase production (Figure 2). CMCase activities of other strains which were not shown in Figure 2 were not detected. The CMCase activity is also approximately consistent with of the results of CMC culture and filter paper culture screening experiment.

### 3.4. Determination of Laccase Activity

The laccase activities of superior strains that had been selected based on Guaiacol color culture screening are shown chronologically in Figure 3. *Coprinus comatus* and *Pleurotus nebrodensis* displayed much higher laccase activities than *Hericium erinaceus* and *Lentinus edodes868*. Laccase activities of *Coprinus comatus* and *Pleurotus nebrodensis* reached their peaks with 496.67 U/L and 489.17 U/L at day 16 and day 18, respectively, while *Hericium erinaceus* reached its peak of 48.54 U/L at day 10 and *Lentinus edodes868* reached its peak of 30.88 U/L at day 11.

*Coprinus comatus* has not only the highest CMCase activity but also the highest laccase activity. It is a kind of delicious mushroom at the same time, so *Coprinus comatus* is a promising mushroom strain to be used in the development of bioenergy.

## 4. Discussion

Cellulose, a homopolysaccharide composed of *β*-D-glucopyranose units, linked by *β*-(1→4)-glycosidic bonds can be converted to cellobiose and glucose by cellulase [4]. Congo red can bind to polysaccharides like cellulose but cannot bind to saccharides to form red compounds. Therefore when cellulose was hydrolyzed to monosaccharide, the unconjugated Congo red was eluted by saline solution and then the transparent circles were apparent in culture plates [20]. If cellulose was not hydrolyzed, it could not bound to Congo red and therefore could not be eluted and remained red. Hankin and Anagnostakis [21] found that transparent circles were produced around the colonies in some cellulose culture medium, while Teather and Wood [22] hold that there, to some extent, existed a linear relationship between the transparent circle size and cellulase activity. However, some strains that were tested in this investigation such as *Lentinus edodes939*, *Lentinus edodes868*, and *Pholiota nameko* showed bigger transparent circles in CMC culture screening but indicated no or low activity in the following CMCase activity test. On the other hand, *Coprinus comatus* with inferior results in CMC culture screening showed the highest CMCase activity in carbomethyl cellulase activity (CMCase) determination. About *d1*/*d2*, the situation was similar. For example, *Coprinus comatus* had the highest CMCase activity and was obviously higher than others, but the *d1*/*d2* of *Coprinus comatus* was inferior. Another case is *Hericium erinaceus*; it had the highest *d1*/*d2*, while CMCase activity was second, but the latter increased remarkably after 9 d during fermentation. In addition, the results of CMC culture and filter paper culture screening experiment are approximately consistent. Therefore, the transparent circle, *d1*/*d2*, degradative degree of filter paper, or CMCase activity alone cannot determine the fungi's ability to secrete cellulase. It is better to combine the results of four aspects to choose the cellulose degrading superior mushroom strains.

Although it indicated that *Coprinus comatus* and *Hericium erinaceus* were preferential candidates of degrading cellulose, it is only under our technique of fermentation. So it is necessary to optimize the liquid culture technique in order to increase the activity of cellulose next stage. And at the same time, the cellulase activity of the strains when they grow on the solid lignocellulose waste is worthy of being studied, because the microorganisms grow under such conditions closer to their natural habitat and can possibly produce larger amounts or higher activity of extracellular enzymes.

In fungi, laccase is known as a ligninolytic enzyme and appears to be involved in fruiting body formation, fungal plant-pathogen/host interaction, and stress defense. Since laccase plays a major role in fungal degradation of lignolytic compounds, its activity to some extent reflects the ability of the fungus to decompose lignocellulosic materials. In addition to a remarkably low substrate specificity, laccase has other properties that make this enzyme potentially useful for biotechnological application, like laccase does not need the addition or synthesis of low molecule weight cofactor. Although laccases have been found in many species, the information from the edible fungi is less reported [23].

In this report, we found that *Coprinus comatus* and *Hericium erinaceus* had good ability of producing CMCase, while *Coprinus comatus* and *Pleurotus nebrodensis* had good ability of producing laccase. So *Coprinus comatus* was able to produce both enzymes at high levels. In recent years, It is found that *Hericium erinaceus* is capable of biodegrading straw [24]. These results may provide a useful tool for screening of high laccase-producing and CMCase-producing strains and offer a fundamental method to study the laccase production capacity of fungi.

As the world energy crisis is becoming increasingly severe, it is necessary and urgent to find a new energy to substitute or complement petroleum energy. Undoubtedly lignocellulose is the optimal candidate. Since microbes play a crucial rule in the process of conversion of lignocellulose to bioenergy, this report may offer a promising mushroom strain to use in the development of bioenergy.

## Figures and Tables

**Table 1:** Ratio of transparent circle to mycelium circle of 10 strains under CMC culture.

Strains	Transparent circle diameter (*d*1)/cm	Mycelium circle diameter (*d*2)/cm	*d*1/*d*2
*Lentinus edodes939 *	3.87 ± 0.22	3.49 ± 0.09	1.11 ± 0.06^C^
*Pholiota nameko *	5.18 ± 0.11	5.04 ± 0.27	1.03 ± 0.06^C^
*Lentinus edodes868 *	4.78 ± 0.22	3.84 ± 0.22	1.24 ± 0.06^C^
*Coprinus comatus *	3.34 ± 0.08	2.42 ± 0.22	1.39 ± 0.17^C^
*Macrolepiota procera *	4.32 ± 0.21	2.32 ± 0.30	1.88 ± 0.27^BC^
*Auricularia auricula *	1.41 ± 0.10	0.62 ± 0.17	2.30 ± 0.30^B^
*Hericium erinaceus *	4.95 ± 0.49	0.61 ± 0.09	8.16 ± 0.56^A^
*Grifola frondosa *	3.84 ± 0.19	1.70 ± 0.11	2.26 ± 0.05^B^
*Pleurotus nebrodensis *	0.00 ± 0.00	2.76 ± 0.51	0.00 ± 0.00^D^
*Shiraia bambusicola *	0.00 ± 0.00	5.91 ± 0.72	0.00 ± 0.00^D^

Different capital letters followed by the data in the table mean the significant difference at 0.01 level.

The data represent the average of three independent experiments.

**Table 2:** The decomposing degree of 10 strains on filter paper.

Strains	The decomposing degree
*Lentinus edodes939 *	++^b^
*Pholiota nameko *	+^c^
*Lentinus edodes868 *	++
*Coprinus comatus *	++
*Macrolepiota procera *	+++
*Auricularia auricula *	++
*Hericium erinaceus *	+++^a^
*Grifola frondosa *	+
*Pleurotus nebrodensis *	++
*Shiraia bambusicola *	0^d^

^a^ + ++ stands for the strain decomposed the filter paper in a good manner.

^b^ + + stands for the strain decomposed the filter paper in a common manner.

^c^+ stands for the strain faintly decomposed the filter paper.

^d^0 stands for the strain decomposed no filter paper.

The data represent the average of three independent experiments.
